# Supplementary material for: Acute Kidney Injury in Patients Undergoing Cardiac Transplantation: A Meta-Analysis
Source: Medicines (Basel). 2019 Nov 1;6(4):108. doi: 10.3390/medicines6040108 (PMC6963309; doi:10.3390/medicines6040108)
Supplement: Supplementary file 1 [file medicines-06-00108-s001.pdf]

medicines

MDPI

Supplementary Materials: Acute Kidney Injury in Patients Undergoing Cardiac Transplantation: A Meta-analysis

Charat Thongprayoon, Ploypin Lertjitbanjong, Panupong Hansrivijit, Anthony Crisafio, Michael A. Mao, Kanramon Watthanasuntorn, Narothama Reddy Aeddula, Tarun Bathini, Wisit Kaewput and Wisit Cheungpasitporn

Study name	Statistics for each study					Relative weight
	Odds ratio	Lower limit	Upper limit	Z-Value	p-Value	
Wyatt et al	3.970	0.953	16.535	1.894	0.058	10.67
De Santo et al	11.250	2.810	45.040	3.420	0.001	11.28
Crudele et al	3.597	0.950	13.620	1.884	0.060	12.25
Fortrie et al	2.270	0.781	6.598	1.506	0.132	19.07
Giglio Canelhas de Abreu et al	2.830	0.911	8.790	1.799	0.072	16.90
Romeo et al	15.870	0.922	273.103	1.904	0.057	2.68
García-Gigorro et al	4.840	1.979	11.836	3.456	0.001	27.15
	4.102	2.574	6.536	5.937	0.000	

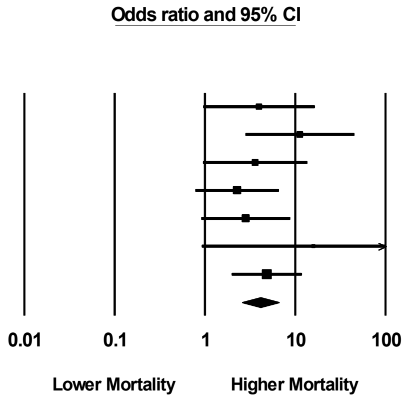

Figure S1. Forest plots of the included studies assessing the pooled OR of hospital mortality among patients undergoing cardiac transplantation with AKI limited to studies with confounder-adjusted analysis.

Study name	Statistics for each study					Relative weight
	Odds ratio	Lower limit	Upper limit	Z-Value	p-Value	
Wyatt et al	3.970	0.953	16.535	1.894	0.058	22.04
Fortrie et al	4.430	1.543	12.722	2.765	0.006	28.58
Romeo et al	44.690	9.089	219.742	4.676	0.000	19.61
García-Gigorro et al	11.030	4.081	29.809	4.733	0.000	29.77
	8.926	3.477	22.915	4.550	0.000	

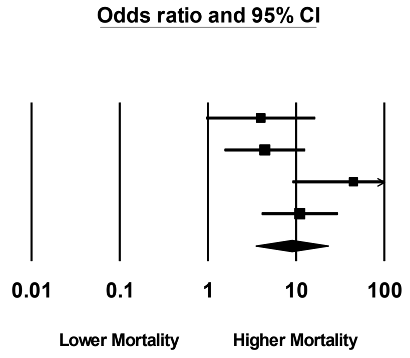

Figure S2. Forest plots of the included studies assessing the pooled OR of hospital mortality among patients undergoing cardiac transplantation with AKI requiring RRT limited to studies with confounder-adjusted analysis.

Study name	Odds ratio	Lower limit	Upper limit	Z-Value	p-Value	Relative weight
De Santo et al	4.250	1.411	12.800	2.572	0.010	11.05
Fortrie et al	2.250	0.930	5.442	1.800	0.072	17.22
Schiferer et al	1.860	1.006	3.438	1.980	0.048	35.59
Hosková et al	1.520	0.422	5.480	0.640	0.522	8.16
Romeo et al	4.440	0.969	20.342	1.920	0.055	5.80
Guven et al	2.180	1.001	4.746	1.963	0.050	22.18
	2.256	1.564	3.255	4.353	0.000	

(Statistics for each study)

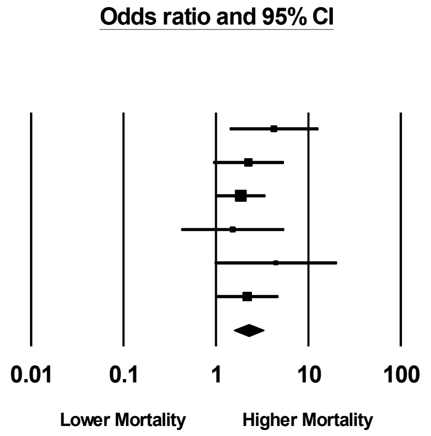

Figure S3. Forest plots of the included studies assessing the pooled OR of 1-year mortality among patients undergoing cardiac transplantation with AKI.

Study name	Odds ratio	Lower limit	Upper limit	Z-Value	p-Value	Relative weight
Ouseph et al	16.670	2.861	97.127	3.129	0.002	6.10
Fortrie et al	2.750	1.135	6.661	2.241	0.025	20.89
Schiferer et al	3.710	1.943	7.082	3.974	0.000	33.75
Romeo et al	6.790	2.248	20.505	3.397	0.001	14.32
Guven et al	2.820	1.277	6.226	2.565	0.010	24.94
	3.889	2.487	6.083	5.953	0.000	

(Statistics for each study)

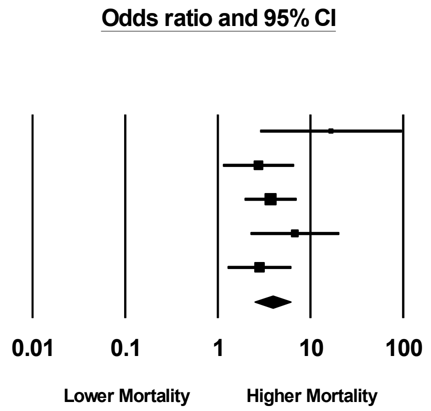

Figure S4. Forest plots of the included studies assessing the pooled OR of 1-year mortality among patients undergoing cardiac transplantation with AKI requiring RRT.

Study name	Odds ratio	Lower limit	Upper limit	Z-Value	p-Value	Relative weight
De Santo et al	4.250	1.411	12.800	2.572	0.010	32.43
Fortrie et al	2.250	0.930	5.442	1.800	0.072	50.55
Romeo et al	4.440	0.969	20.342	1.920	0.055	17.02
	3.105	1.657	5.817	3.536	0.000	

(Statistics for each study)

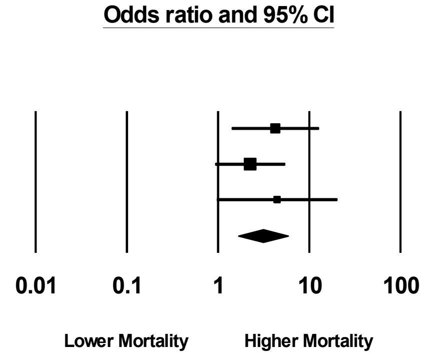

Figure S5. Forest plots of the included studies assessing the pooled OR of 1-year mortality among patients undergoing cardiac transplantation with AKI limited to studies with confounder - adjusted analysis.

Study name	Odds ratio	Lower limit	Upper limit	Z-Value	p-Value
Fortrie et al	2.750	1.135	6.661	2.241	0.025
Romeo et al	6.790	2.248	20.505	3.397	0.001
	4.057	1.688	9.751	3.129	0.002

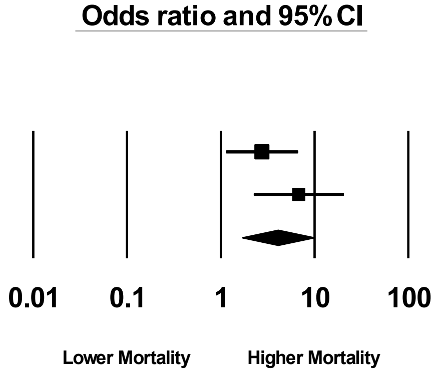

Figure S6. Forest plots of the included studies assessing the pooled OR of 1-year mortality among patients undergoing cardiac transplantation with AKI requiring RRT limited to studies with confounder-adjusted analysis.

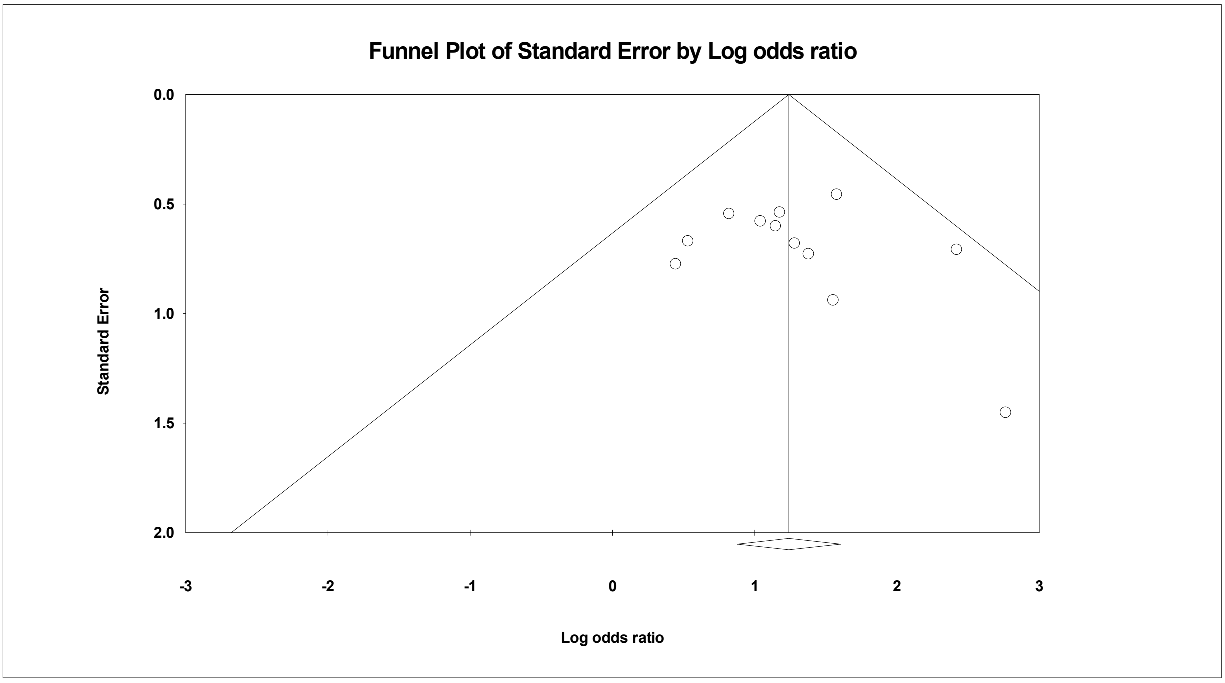

Figure S7. Funnel plot evaluating for publication bias evaluating hospital mortality (and/or 90-day mortality) of AKI among patients undergoing cardiac transplantation.

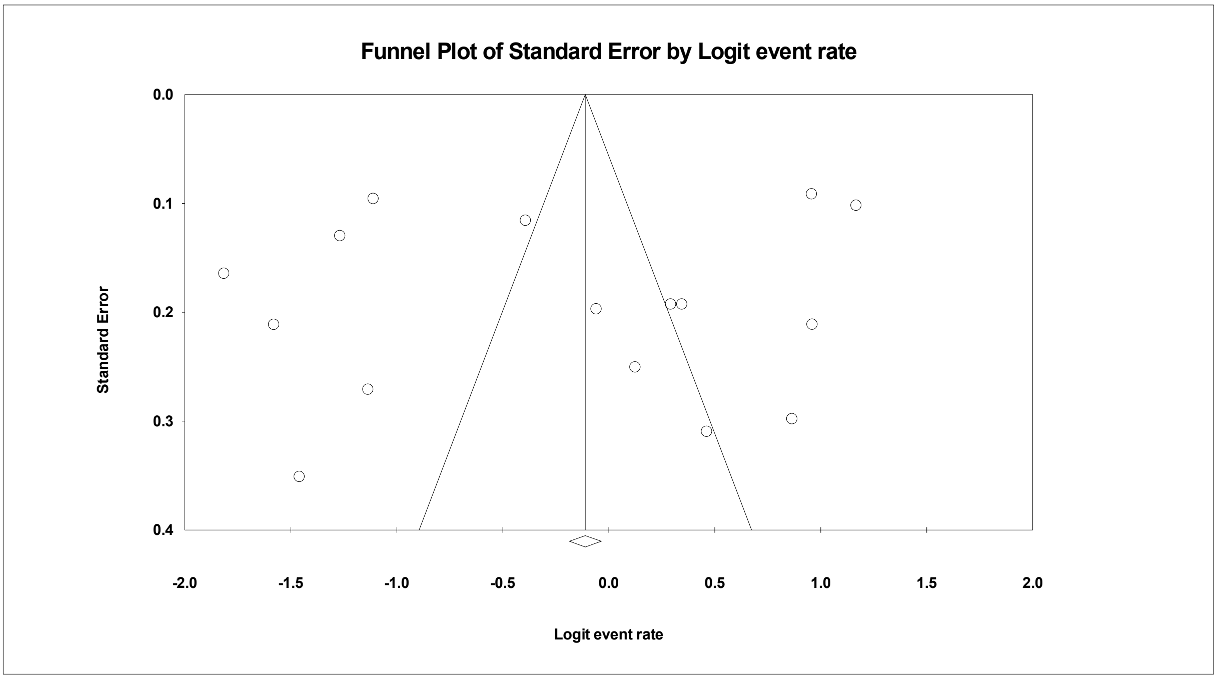

Figure S8. Funnel plot evaluating for publication bias evaluating 1-year mortality of AKI among patients undergoing cardiac transplantation.

Database: Ovid MEDLINE

1. exp acute kidney injury/
2. acute kidney injury$.mp
3. exp acute renal failure/
4. acute renal failure$.mp.
5. exp renal insufficiency/
6. renal insufficiency$.mp.
7. exp dialysis/
8. dialysis$.mp.
9. hemodialysis$.mp.
10. renal replacement therapy$.mp.
11. hemofiltration$.mp.
12. hemodiafiltration$.mp.
13. 1 or 2 or 3 or 4 or 5 or 6 or 7 or 8 or 9 or 10 or 11 or 12
14. heart.mp
15. cardiac.mp
16. 14 or 15
17. Transplant.mp
18. Transplantation.mp
19. 17 or 18
20. 16 and 19
21. 13 and 20

Database: EMBASE

('heart transplantation' OR 'heart graft' OR 'cardiac graft rejection' OR 'patient history of heart transplantation') AND 'acute kidney failure'

Cochrane Database

"heart transplantation" AND "acute kidney injury"
